# Processes and dynamics of linkage to care from mobile/outreach and facility-based HIV testing models in hard-to-reach settings in rural Tanzania. Qualitative findings of a mixed methods study

**DOI:** 10.1186/s12981-018-0209-8

**Published:** 2018-11-20

**Authors:** Erica S. Sanga, Ferdinand C. Mukumbang, Adiel K. Mushi, Willyhelmina Olomi, Wondwossen Lerebo, Christina Zarowsky

**Affiliations:** 1 NIMR-Mwanza Medical Research Centre (MMRC), Mwanza, Tanzania; 2 School of Public Health, University of Western Cape, Cape Town, South Africa; 3 School of Public Health, Mekelle University, Mekelle, Ethiopia; 4 National Institute for Medical Research (NIMR), Dar-es-Salaam, Tanzania; 5 University of Montreal Hospital Research Centre and School of Public Health, Université de Montréal, Montreal, Canada; 6 Department of Public Health, Institute of Tropical Medicine, Antwerp, Belgium; 7 NIMR-Mbeya Medical Research Centre (MMRC), Mbeya, Tanzania

**Keywords:** HIV, Linkage to care, Health systems integration, Mixed methods process evaluation, Mobile/outreach HIV testing, Rural health, Hard to reach populations, Stigma, Mbeya, Tanzania

## Abstract

**Background:**

Like other countries, Tanzania instituted mobile and outreach testing approaches to address low HIV testing rates at health facilities and enhance linkage to care. Available evidence from hard-to-reach rural settings of Mbeya region, Tanzania suggests that clients testing HIV+ at facility-based sites are more likely to link to care, and to link sooner, than those testing at mobile sites. This paper (1) describes the populations accessing HIV testing at mobile/outreach and facility-based testing sites, and (2) compares processes and dynamics from testing to linkage to care between these two testing models from the same study context.

**Methods:**

An explanatory sequential mixed-method study (a) reviewed records of all clients (n = 11,773) testing at 8 mobile and 8 facility-based testing sites over 6 months; (b), reviewed guidelines; (c) observed HIV testing sites (n = 10) and Care and Treatment Centers (CTCs) (n = 8); (d) applied questionnaires at 0, 3 and 6 months to a cohort of 1012 HIV newly-diagnosed clients from the 16 sites; and (e) conducted focus group discussions (n = 8) and in-depth qualitative interviews with cohort members (n = 10) and health care providers (n = 20).

**Results:**

More clients tested at mobile/outreach than facility-based sites (56% vs 44% of 11,733, p < 0.001). Mobile site clients were more likely to be younger and male (p < 0.001). More clients testing at facility sites were HIV positive (21.5% vs. 7.9% of 11,733, p < 0.001). All sites in both testing models adhered to national HIV testing and care guidelines. Staff at mobile sites showed more proactive efforts to support linkage to care, and clients report favouring the confidentiality of mobile sites to avoid stigma. Clients who tested at mobile/outreach sites faced longer delays and waiting times at treatment sites (CTCs).

**Conclusions:**

Rural mobile/outreach HIV testing sites reach more people than facility based sites but they reach a different clientèle which is less likely to be HIV +ve and appears to be less “linkage-ready”. Despite more proactive care and confidentiality at mobile sites, linkage to care is worse than for clients who tested at facility-based sites. Our findings highlight a combination of (a) patient-level factors, including stigma; and (b) well-established procedures and routines for each step between testing and initiation of treatment in facility-based sites. Long waiting times at treatment sites are a further barrier that must be addressed.

## Background

In Tanzania, an estimated 1.5 million people were living with HIV in 2013, a 5.1% prevalence amongst the adult population [1, 2]. HIV testing and successful linkage of people with HIV into HIV care constitute important first steps in effective management of HIV/AIDS, and in reaching the 90–90–90 goal set by the WHO: 90% of people with HIV infection are diagnosed, 90% of those diagnosed start ART, and 90% of those taking ART achieve viral load suppression by 2020, and the goal of an AIDS-free world by 2030 [3, 4]. In the context of low levels of HIV testing in Tanzania [5–7], the government promoted out-of-facility-based testing services to complement health facility-based voluntary counselling and testing (VCT), which predominantly existed alone until the early 1990s [8–10]. The out-of-facility-based testing (referred to as the “mobile/outreach testing model” in this study) currently recommended in the country includes home-based testing, workplace or school-based testing, and testing through mobile vans [7, 10, 11]. HIV testing coverage is improving; the 2011–2012 Tanzania HIV and Malaria Survey showed that 90% of Tanzanians know where to get an HIV test and 67% of women and 50 per cent of men have ever been tested for HIV. The HIV epidemic in Tanzania is largely generalized though with concentrated epidemics among key populations such as mobile populations, people who inject drugs, men who have sex with men and sex workers. However heterosexual sex accounts for over 80% of all HIV infections in Tanzania [12].

### Linkage to care

Regardless of how people get to know their HIV status, what is of primary importance is that those who test HIV-positive be successfully linked to HIV treatment and care for further medical care, psychological and social support [7, 13]. Linkage to care has been described as the process of assisting individuals with an HIV positive diagnosis to enter into HIV medical care, or the process of engaging newly-diagnosed HIV-infected persons into HIV primary care [14–16]. It is a crucial step along the HIV continuum of care for better management and prognosis for HIV-positive individuals [17–19]. Linkage to care has been defined as attending at least one clinic appointment within 90 days following diagnosis [15, 20].

Other studies defined linkage to care as attending one or more clinic visit for HIV care within 1–6 months of diagnosis, or more than two visits within 6–12 months of diagnosis [14, 21]. In this study, we operationalized linkage to care as attending and completing the first step of registration at the HIV care clinic and receiving an HIV care and treatment card within the first 6 months after diagnosis [21, 22]. Studies conducted in South Africa reported 72% and 61% of clients testing at facility or clinic-based HIV testing sites were linked to care within 6 months of diagnosis [23, 24]. In Tanzania, a study conducted in Kisesa, a rural area in Mwanza region, showed a linkage of 14% at 4 months after HIV diagnosis from the facility and community-based modalities [25].

Tanzania’s national HIV testing and counselling guidelines (2013) stipulate that after completion of HIV testing, all HIV-positive individuals should be linked to receive appropriate care and treatment services at designated care and treatment centres (CTCs) [6, 7]. To this end, all HIV testing sites in Tanzania are expected to establish referral links to these centres [6, 7]. However, it has been reported that while mobile/outreach HIV testing and counselling (HTC) models are now widely used in Africa, the rates of linkage to care and initiation of antiretroviral therapy (ART) are often low [13, 26, 27]. In Tanzania, like other sub-Saharan African countries, poor referral systems, transport, poor clinic organization, and inadequate resources pose continuing challenges to linkage to care [9, 25, 28]. In addition, the clients receiving testing at mobile sites may differ from those testing at facilities. We recently reported a two-armed cohort study which found that clients testing HIV-positive at facility-based sites were more likely to link to care than those testing at mobile/outreach sites [29]. This paper seeks to understand this outcome.

This paper’s two objectives are (1) to describe the populations accessing HIV testing at mobile/outreach and fixed/facility-based testing sites in hard-to-reach rural settings of Mbeya region, Tanzania, and (2) to compare processes and dynamics from testing to linkage to care between these two testing models.

## Methods

**Fig. 1:**
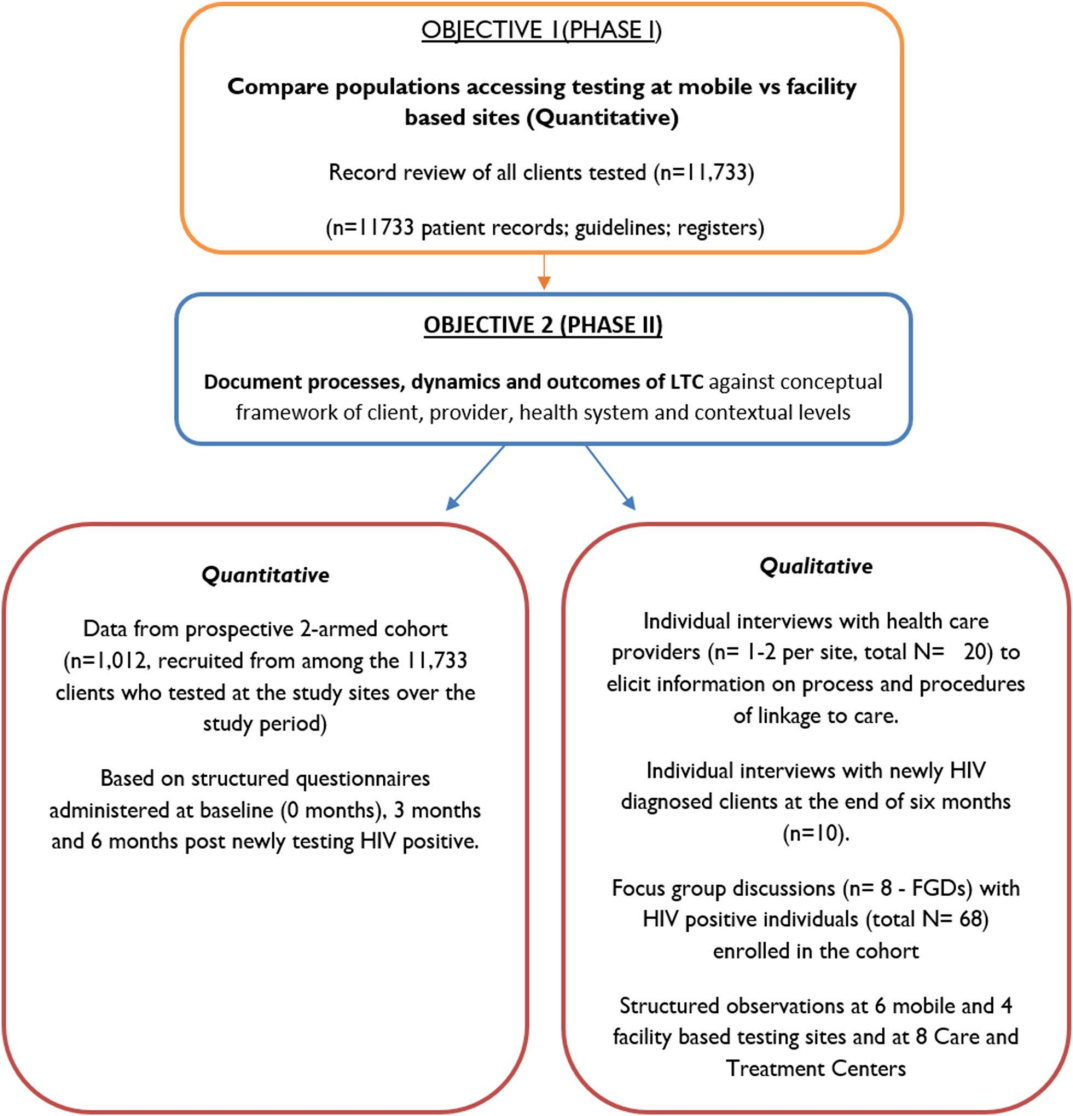
Methods framework

This was an explanatory sequential mixed-method study, where qualitative methods were used to complement the quantitative results [30] in a process evaluation approach [31], which helped us to (1) compare the populations accessing the two models of testing; (2) describe the linkage to care models; and (3) explore and compare the actual practices by staff and intended users [31], in order to shed light on the primary outcomes of the cohort study (rates and timelines of linkage to care between the two models) [37]. The study reviewed guidelines and records (register books and reports) at 16 mobile and fixed facilities (8 + 8), to complement a two-armed cohort study of 1012 newly-diagnosed HIV-positive individuals recruited from these same sites and interviewed with structured questionnaires on HIV testing and linkage to care at 0, 3 and 6 months after diagnosis. We observed selected sites and conducted eight focus group discussions and ten in-depth interviews with newly HIV diagnosed individuals enrolled in the cohort study, and individual interviews with healthcare providers (n = 20) working at either at HIV testing units or CTCs. Sixty-eight HIV-positive individuals participated in eight focus group discussions (FGDs) each with six to 12 respondents. This paper reports on analyses of additional data from the cohort study to interpret findings previously reported [29] and adds new findings on populations accessing the two models of HIV testing and on processes and dynamics of linkage to care. Figure 1 outlines the methods for the objectives reported here.

### Sampling

Multiple sampling techniques were applied to select the study sites and respondents from four districts in Mbeya region, Southern Tanzania. Districts were purposively selected to represent hard-to-reach populations, including two districts on the highway to Malawi and Zambia, and two remote districts. In total, 16 study sites were randomly selected. These included two facility-based and two mobile/outreach testing sites in each district. Though only a minority of health facilities in the region offer Care and Treatment services, all facility-based sites randomly selected for this study were in facilities which also had CTCs.

We reviewed records for all individuals receiving HIV testing services at the study sites between August 2014 and February 2015 (n = 11,733), covering the period during which a cohort of 1012 HIV-positive participants had been recruited from the study sites. Respondents for qualitative interviews were purposively selected from the main cohort of adults above 18 years having tested HIV-positive at the selected facility-based and mobile/outreach sites. Details of cohort construction and recruitment have been reported elsewhere [37].

The study had enrolled 1012 HIV-positive participants evenly divided across the two arms of the study (testing at mobile/outreach vs facility-based HIV testing sites). Cohort retention at 6 months was 83% overall, 87% in the facility-based testing arm and 76% in the mobile/outreach-testing arm. Health workers were purposively selected to include nurse counsellors at HIV testing sites, nurse counsellors and doctors at the care and treatment centre, and in three sites we interviewed the site in-charge. One day of structured non-participant observation was done by the lead investigator (ESS) at each of the study sites indicated in Table 1, with an assistant at some sites, taking notes of all ongoing activities related to either HIV testing or HIV care. In addition, about 32 h were spent at eight care and treatment centres observing actual registration and linkage to care activities. The researcher and the assistant compared and compiled their respective notes and observation guides after each day of observation.

**Table 1:** Research sites and data collection methods

Districts	Type of site	IDI-HCP	Observation/field- notes	**IDI-client**	**FGDs**
Facility-based sites					
Kyela	Kyela hospital	x	x	x	
	Ipinda health centre	x	x	x	X
Mbozi	Vwawa hospital	x	x		
	Tunduma health centre	x	x	x	X
Mbeya rural	Ifisi hospital	x	x	x	X
	Inyala health centre	x	x		
Chunya	Chunya hospital	x	x	x	X
–	Makongolosi dispensary	x	x		
Mobile/outreach sites					
Kyela	ST JOHN HUS-Kyela	x	x	x	X
	MMRC mobile-Kyela	x	x	x	
Mbozi	SHDEPHA-Mpemba	x	x	x	
	MMRC mobile-Mbozi	x			X
Mbeya rural	KIHUMBE-Mbalizi	x	x	x	X
	MMRC mobile-Mbeya Rural	x			
Chunya	KIHUMBE-Chunya	x	x	x	X
	MMRC-mobile-Chunya	x	x	x	X

### Data collection procedures

We recruited cohort participants between August and December 2014, and follow-up questionnaire administration continued until July 2015. Participant enrollment was done at the selected study sites with the assistance of the nurse counsellors who introduced the research team to clients during health education sessions. The research team explained the study briefly, and interested individuals were invited to a private room for detailed explanation, informed consent and agreement at a convenient time for questionnaire administration. Data were collected by research assistants who underwent a 2-day training on protocol, informed consent, and data collection procedures. Data collection tools were pretested in two sites not selected for this study.

Questionnaire administration, document review and records/register review was done in all 16 selected study sites, while site observation/field notes, health care providers in-depth individual interviews (IDI), client in-depth individual interviews, and focus group discussions (FGDs) were conducted in some of the sites, as shown in the table below (Table 1). Qualitative interviews and FGDs were conducted in Swahili by the lead investigator (ESS), assisted by four trained research assistants.

### Data analysis

Quantitative data from sites were recorded, cleaned and analyzed using Stata Version 13 (College Station Texas, USA). Descriptive analysis methods were used to present the characteristics of participants (frequencies and percentage for categorical data, measures of central tendency, and dispersion). Cross-tabulation was used to show the distribution of study subjects by the testing site where we had multiple possible categories. Statistical significance was declared at P-values < 0.05.

Qualitative interview data were audio recorded and transcribed verbatim in Swahili. The transcripts were translated by two professional translators to English, and each transcript was checked by the first author. The transcripts were analyzed using Atlas.ti version 7, a qualitative data management software [32]. The data were analyzed by ESS and FCM using the thematic content analysis approach [33]. We employed the following steps: (1) free coded the data; (2) identified themes and subthemes; and (3) selected themes that are important for this study. Observational data were analyzed against a grid developed to assess adherence to national guidelines and the elements of the conceptual framework guiding the study and were further triangulated against questionnaire and qualitative interview data.

Our conceptual framework was developed on the basis of existing frameworks and literature on access to care. The conceptual framework included individual, provider, health system and contextual factors [13, 22, 34, 35]. We also compared the observed and reported HIV testing and linkage to care processes against the Tanzanian national guidelines [7, 36].

### Rigour and trustworthiness

To enhance rigour and trustworthiness, we used multiple HIV testing sites and triangulated three different data collection methods with a variety of respondents after piloting, as described above. Research assistants underwent an intensive training for quality assurance. Data analysis was conducted by two of the authors and crosschecked by the last author. Finally, we followed guidelines for reporting qualitative research [37].

### Ethical considerations

The study received ethical clearance from The University of Western Cape (UWC) Senate Research Committee, the Mbeya Medical Research Centre, the Mbeya Medical Research Ethics Committee (MMREC) and the National Health Research Ethics Sub-Committee (NatHREC), under the Tanzanian National Institute for Medical Research (NIMR). Prior to data collection at each clinic, the research team briefed the nurse counsellors about the study objectives and procedures. Interested individuals were invited to a private room for detailed explanation, informed consent process and agreement on a convenient time and place for the questionnaire to take place. Participation in the study was voluntary, and it was explained to participants that they were free to withdraw from the study at any time without negative consequences. Volunteers were provided with an information sheet containing all details about the study. They signed an informed consent form, and confidentiality procedures and anonymity were observed.

## Results

We first compare the overall client base of the sites comprising the two testing models (Table 2). Next, we compare the two models in terms of the processes and dynamics from HIV testing to linkage into care, subsequently analyzing each approach with regard to the individual, healthcare provider, health system, and contextual levels.

**Table 2:** Characteristics of clients tested at facility vs. mobile/outreach sites over the period of six months (N = 11,733)

Variable	Facility-based (n = 5234)	Mobile (n = 6539)	*P* value
Age group (years)			
< 25	1395 (26.7)	2045 (31.3)	P < 0.001
25–39	2389 (45.6)	2756 (42.2)	
40–59	1147 (21.9)	1360 (20.8)	
> 60	303 (5.8)	378 (5.7)	
Age mean (SD)	33.82 (18.7)	33.06 (13.3)	
Gender			
Male	2261 (43.2)	3066 (46.9)	P < 0.001
Female	2973 (56.8)	3473 (53.1)	
Marital status			
Single	1363 (26.1)	2063 (31.6)	P < 0.001
Married	3107 (59.6)	3787 (57.9)	
Separated	379 (7.3)	289 (4.4)	
Divorced	34 (0.7)	87 (1.3)	
Widow	331 (6.4)	308 (4.7)	
Education			
None	160 (3.7)	561 (8.8)	P < 0.001
Primary	3784 (88.1)	4944 (77.6)	
Secondary	304 (7.1)	700 (10.9)	
Post-secondary	45 (1.1)	165 (2.6)	
Occupation			
Unemployed	125 (2.9)	91 (1.4)	P < 0.001
Employed	78 (1.8)	173 (2.7)	
Self employed	3946 (92.1)	5882 (92.3)	
Student	138 (3.2)	228 (3.6)	
Ever tested HIV			
No	3064 (58.5)	3236 (49.5)	P < 0.001
Yes	2170 (41.5)	3303 (50.5)	
Results			
Positive	1123 (21.5)	516 (7.9)	P < 0.001
To whom planned to disclose			
Spouse/partner	2758 (52.7)	3540 (54.1)	P < 0.001
Relatives	2291 (43.8)	2572 (39.3)	
Others	153 (2.9)	411 (6.3)	
No plan to disclose	32 (0.6)	16 (0.3)	
To whom planned to disclose (HIV positive client)			0.014
Spouse/partner	640 (56.9)	282 (54.7)	
Relatives	459 (40.9)	215 (41.7)	
Others	15 (1.3)	18 (3.5)	
No plan to disclose	9 (0.8)	1 (0.2)	

### Description of individuals tested for HIV at the facility-based and mobile/outreach sites during the study period

We reviewed registers from the 16 study sites to collect information related to all individuals attending the sites for HIV testing services, including but not limited to the cohort of 1012 newly HIV diagnosed participants. A total of 11,733 individuals received HIV testing services during the study period. More people tested at the mobile/outreach sites than facility-based sites (56% vs 44%, p < 0.001)). More women (55%) attended HIV testing services than males; and the mean age was 33 years, with most clients being married and self-employed in small-scale businesses. Table 2 describes the characteristics of the clients testing in the two models. Chi Square test showed statistically significant differences in patients’ characteristics between facility-based and mobile testing sites, notably that clients testing at mobile/outreach sites were slightly younger (p < 0.001) and more likely to be male (p < 0.001), and that the proportion of clients testing HIV positive was significantly higher at the facility-based sites than at mobile/outreach sites (21.5% vs 7.9%, p < 0.001). We were unable to determine how many clients were linked into care because the registers are filled in immediately after testing and counselling and contained no information on linkage status. However, site staff at all sites reported verbally that all clients tested positive were provided with a referral form to bring to the CTC of their choice. It was also noted that the referral form has a section at the end of the form that was supposed to be cut off by the nurse at the CTC and brought back to the HIV testing section for feedback to the counsellor, but in most sites there was no evidence that these had been brought back to the HIV testing section.

### Mobile (Outreach) HIV testing model: processes and dynamics

Mobile sites refer to HIV testing services outside of regular health facilities. In this study, they included stand-alone single-purpose, HIV testing sites operating under different NGOs such as Walter Reed Program, Mbeya Medical Research centre, USAID, PACT Tanzania, SHDEPHA Mbeya HIV network, KIHUMBE, and Faith-Based Organization such as the Anglican and Moravian missions. The mobile/outreach teams move from one place to another in the community or work in venues outside of public sector healthcare facilities.

The mobile or outreach HIV testing services include but are not limited to: campaigns; mobile testing clinics using cars or tents; home visits; and workplace, school or special event testing services like World AIDS Day. Most of the mobile/outreach testing sites are coordinated by Non-Governmental Organizations that work in collaboration with the Mbeya Regional Medical Office and the Ministry of Health. All mobile/outreach sites included in the study were working under NGOs and were funded by different organizations, such as the Henry Jackson Foundation through Walter Reed program, USAID, the European & Developing Countries Clinical Trials Partnership (EDCTP) and church organizations. All eight mobile sites had register books for individuals coming for HIV testing, as well as monthly and annual reports in place. The mobile/outreach testing sites had more complete records of the clients attending care in their sites compared to public facility based sites.

### Processes and procedures at the mobile/outreach sites

Our observations and respondent reports revealed that all mobile/outreach sites offer HIV testing, and then refer HIV-positive clients (with a referral letter) to the nearby CTCs for further care. One mobile site from the Mbeya Medical Research Centre offers CD4 testing and HIV staging in addition to HIV testing, then refers the HIV-positive individuals to the nearest CTC. In some of the mobile sites, we observed the care providers assisting the newly-diagnosed client’s link into care by escorting them to the CTCs, or by visiting them at home to encourage them to seek HIV care services.

We observed larger numbers of individuals waiting for HIV testing before opening hours in the outreach services compared to the number of clients at the facility-based services. During focus group discussions and individual interviews, when asked the reason for choosing the outreach site for HIV testing, a significant number of respondents reported that they heard announcements when passing by, or they saw the HIV testing truck parking and, seeing many people around, they came and decided to test. Ten clients said they feel more comfortable testing at the mobile site for confidentiality reasons because the service providers in the mobile teams do not come from their community:

*I have always been scared to test for HIV for a long time, when I heard that there are specialists from Mbeya town offering free HIV test and other tests. I said let me go and test because no one knows me in that team so even if I am positive nobody in this community will know that I have this[HIV] problem*[Female 30 yrs.].

Some participants responded that the entertainment taking place during outreach services and campaigns are what motivated them to come closer, and then they decided to test for HIV.

In the focus group discussions about processes of linkage to care, some clients who tested at the mobile sites expressed their wish that the mobile/outreach site could also offer ART, instead of referring them to the facility-based sites:

*The nurse gave me a letter, she said I should come to… hospital for CD4 test probably I will need ART since I was very weak, the day we went to the hospital there were so many people and the queue was long, I said if KIHUMBE* (the site where she tested) *was also providing medication (ART) it would have saved me from this distress* [FGD_5_].

In the mobile/outreach testing sites, the number of clients would vary depending on the type of activity. For example, at an HIV testing campaign or special events like World AIDS Day, where normally there are advertisements and car announcements and posters inviting people to come to receive free HIV testing, up to 200 people are testing for HIV daily. At these events, there are 2–6 nurse-counsellors, 1–2 doctors and 2–4 other support staff, including recorders, HIV health educators, and drivers. However, when these sites conduct HIV testing at their office (stand-alone VCT site) or on home visits, the number of clients was 20–60 per day, with 2-4 staff on duty. Of the 405 study participants who tested at the mobile sites, 69% (95% CI 0.65% to 0.74%) successfully linked to HIV treatment and care in a qualified CTC within 6 months [38].

### Facility-based HIV testing model: Processes and dynamics

In this study “facility-based sites” refers to fixed or static facilities within the public or mission health sector, like hospitals, health centres, or dispensaries where individuals walk in for HIV testing, or where they came for other illnesses but the care providers advise them to take an HIV test as well. Although only 21% of facilities in Mbeya qualify as CTC’s, all eight randomly selected facility-based sites had HIV testing services and CTCs within the campus.

### Processes and procedures at the facility-based site

The eight facility-based sites in this study had an HIV testing section/unit and a physically separate HIV care section (CTC) within the same campus. Therefore, clients testing HIV positive in these sites also had to be linked to a care and treatment centre for further HIV care services, with a referral letter that had to be taken to the nurse at the CTC section, where the client will register into care. Normally the clients go to the CTC on their own unless there is a situation that needs to be explained to the CTC care provider. For example, if the person is not from the area (was visiting family or friend), they should enter care in their home area, but if they currently have a medical condition that needs doctor’s attention, the HIV testing nurse can escort the client to the CTC. At that point, s/he will be given a card with their CTC number that can be linked to the client’s file that is stored at the clinic.

The sites had register books for individuals coming for HIV testing or/and coming for entry into HIV care (registration at CTC). However, some sites had incomplete documentation, with missing information about individuals attending HIV testing and HIV care in the register books.

Qualitative interviews revealed that some of the individuals who tested at the facilities did not plan in advance to be tested. For instance, they went to the clinics because they felt unwell and as part of the screening and diagnosis, the health care providers suggested an HIV test. In some instances, these individuals had been referred from lower-level health facilities such as dispensaries because they had severe health problems that required management at higher-level facilities (district level hospital). Through PITC, these individuals were advised to take an HIV test. Similarly, clients who were admitted to the facility for other illnesses got an HIV test as part of management. PITC is done at the outpatient department as well as in the patient’s wards, although nurses in the ward may refer the patient to the HIV testing section if the ward does not have a qualified HIV counsellor. When an individual tests HIV positive, s/he is provided with a referral letter/form and the client then proceeds to the designated CTC.

### Linkage to care at care and treatment centres: processes and dynamics

Once referred to a CTC, clients testing positive either at facility-based sites or at mobile/outreach sites bring a referral letter to the designated CTC, generally in another part of the facility for facility-based sites. The client hands the referral letters obtained from the testing sites to the nurse responsible for registering newly-diagnosed HIV-positive individuals. The individuals’ details are recorded in a register, and the individuals are provided with a clinic card (CTC-card) detailing their personal information and clinic appointment visits. They have to bring this card every time they attend their clinic or go to any HIV care clinic in the country. The whole procedure of registration takes between 1 and 3 h including waiting time. When this process is complete, the individual is considered to have entered or linked to HIV care. By the end of the study, linkage to care analysis showed that 84% (95% CI 0.81–0.87%) of participants tested at the facility-based testing sites were linked into care within the first 6 months of HIV diagnosis, significantly higher than the 69% linked from the mobile sites (p < 0.001) [38].

With the individual successfully linked to care, they are sent to a clinician for HIV staging, a prescription for CD4 count test, and TB screening (clinicians include medical doctors, assistant medical officers, and clinical officers, all of whom can prescribe or write laboratory requisition in Tanzania). In some sites, the client goes straight to CD4 testing if the services are available on the same day. However, most clinics only offer CD4 testing on selected days of the week, meaning that if their doctor consultations fall on a day that the CD4 testing services are unavailable, the clients are told to return on a day the services are available. Clients who had tested at the single research mobile laboratory which included CD4 count testing did not have to repeat a CD4 count; the doctors would make care decisions based on the result the client brought from the testing site. In most cases, CD4 results were available within 3–7 days and clients had to return to the CTC for these results and proceed to the next step: the decision over whether to be started on ART or not.

Every HIV-positive individual is required to attend treatment adherence sessions for at least 3 days. In some of the sites, the individual could have the adherence sessions while awaiting their lab results. At certain sites, these adherence-counselling sessions are organized for three consecutive days, while at other treatment centres, the adherence counselling sessions are done on the same day each week for three consecutive weeks.

When the CD4 test results are received from the laboratory, the clinician makes the decision of whether or not to start the individual on ART. If the individual has a CD4 count of less than 350 cells, they are initiated on ART. Three hundred and fifty (350 cell) was the cut-off point during the time of data collection; however, recently (2016) there has been a circular from the Ministry of Health on HIV treatment, implementing the ‘Test and treat’ approach, regardless of the CD4 count. The process for the ART-eligible clients to be initiated from the day of registration takes between one and 3 weeks. By the end of the study, 793 (78%) of the participants were enrolled in CTCs. Among those who were linked into care, 77% were initiated on ART. Participants tested in the facility-based sites were initiated on ART faster than those tested in the mobile site. In the first week since registration, 27 (39%) of those tested in a facility were on ART, compared to 19 (37%) tested in mobile sites. The majority of participants (92%) attended between 3 and 5 clinic visits/appointments before starting ART (Table 3).

**Table 3:** Process/procedures and timelines for a newly diagnosed client at the CTC

Variables	Total	Facility-based	Mobile	Chi square
	N (%)	N (%)	N (%)	P value
Time to complete registration and other procedure on the first day				
<1 h	5 (0.6)	3 (0.59)	2 (0.71)	P < 0.001
1–3 h	388 (48.9)	291 (56.8)	97 (34.5)	
3–5 h	334 (42.12)	201 (39.26)	133 (47.33)	
> 5 h	54 (6.8)	12 (2.34)	42 (14.95)	
NA	12 (1.5)	5 (0.98)	7 (2.49)	
Give blood for the CD4 count on same day as registration				
Yes	455 (57.4)	278 (54.3)	177 (62.9)	0.006
No	326 (41.1)	229 (44.7	97 (34.5)	
NA	12 (1.5)	5 (0.98)	7 (2.49)	
Time takes to receive CD4 results				
Same day	13 (1.6)	11 (2.2)	2 (0.7)	0.18
2–7 days	742 (93.6)	479 (93.55)	263 (93.59)	
8–14 days	26 (3.28)	17 (3.32)	9 (3.2)	
NA	12 (1.5)	5 (0.98)	7 (2.49)	
Started ART				
Yes	613 (77.3)	402 (78.5)	211 (75.1)	0.28
No	180 (22.7)	110 (21.5)	70 (24.9)	
How regularly do you check CD4				
Every 3 months	495 (69.4)	345 (67.4)	150 (53.4)	P < 0.001
Every 6 months	298 (32.6)	167 (32.6)	131 (46.6)	
The time it takes to start ART from registration				
1–7 days	234 (38.2)	157 (39.1)	77 (36.5)	P < 0.001
8–14 days	234 (38.2)	174 (43.3)	60 (28.4)	
15–30 days	93 (15.2)	43 (10.7)	50 (23.7)	
30–90 days	39 (6.4)	23 (5.7)	16 (7.6)	
> 90 days	13 (2.1)	5 (1.2)	8 (3.8)	
Visit before starting ART				
1–2 visits	38 (6.8)	13 (3.5)	25 (13.5)	P < 0.001
3–5 visits	519 (92.4)	363 (96.3)	156 (84.3)	
6–10 visits	5 (0.9)	1 (0.3)	4 (2.2)	

**Fig. 2:**
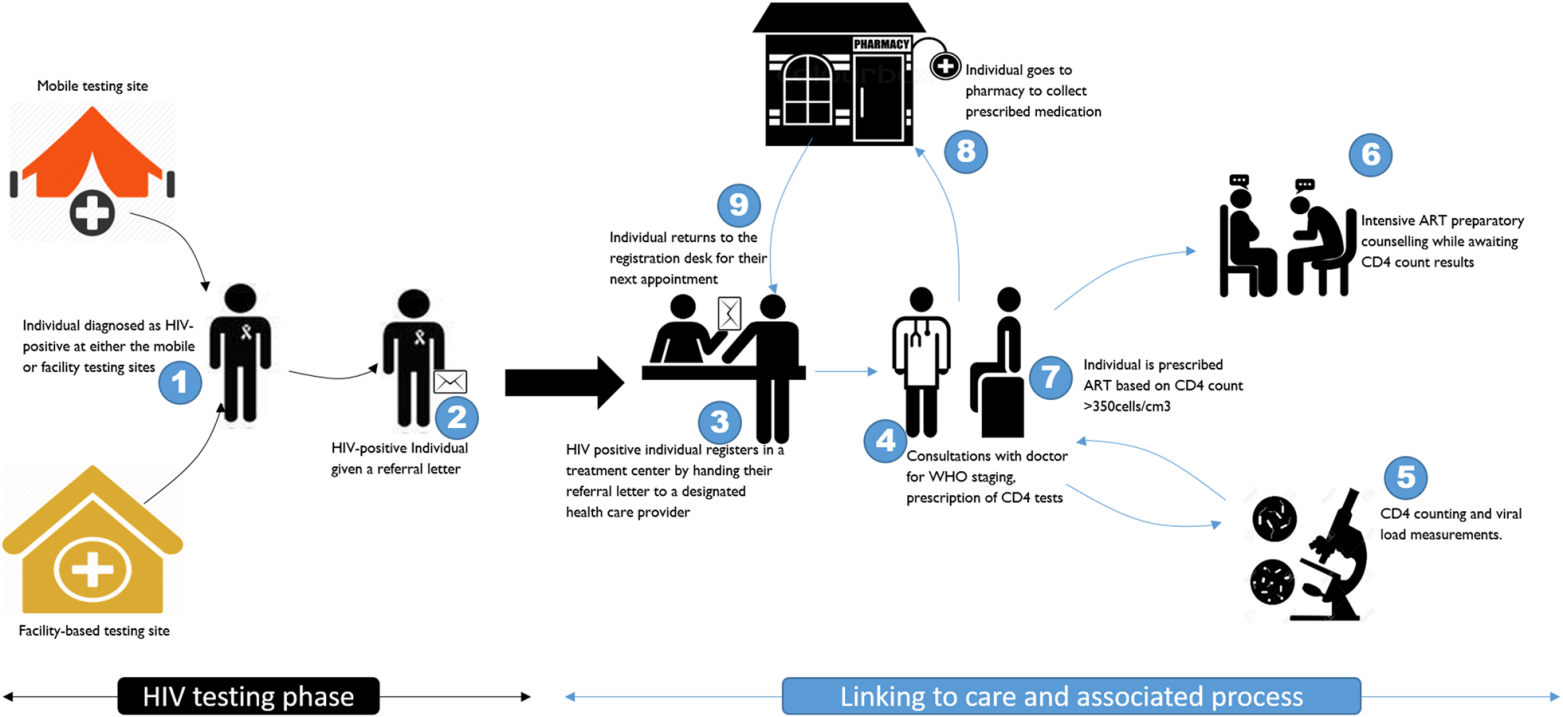
A diagrammatic illustration of the linkage to care process

With a CD4 count above 350, individuals were not eligible for ART at the time of this study, unless they had other conditions like TB, or were in HIV stage III and IV. Non-eligible individuals were given an appointment to return after 3 months, for continuous follow-up and checking of ART eligibility. In some facilities, it is highly recommended that each individual should share their status with a significant other (relative or a friend) to have them as a treatment supporter (accompagnateur). In other sites, this recommendation was not strongly emphasized. Finally, the individual is sent to the pharmacy for their medication to be dispensed. After receiving the medication, the individual returns to the nurse at the registration desk to get the date of their next appointment (Fig. 2).

### Estimated number of clients and staff at the CTC per day

The number of clients at the CTC was high in all sites that offered HIV care and treatment services: between 60 and 120 clients per day in most sites, and more in some sites on some days. In the facility-based sites, the HIV testing sections did not have long queues of people waiting for services. Most of the facilities tested about 10–30 clients per day. In most facility-based testing sites, there were one or two nurse counsellors at the HIV testing station. In all facilities, the section of care and treatment services had 2–6 Nurses, 1–3 Doctors and 2–5 other cadres like lab technicians, pharmacist, recorder etc. Some sites also had Home Based Carers (HBCs) or stable patients in ART known as treatment experts, who were assisting with giving health education on treatment adherence or organizing patients’ files. In the health centres and dispensaries, we observed 50–100 clients in the waiting area. These clients are expected to be seen by 2–3 nurses and 1–2 doctors. For this reason, the waiting times at the waiting areas could extend to 6 h. This was also reported by health care providers and clients during the in-depth interviews.

Whilst the actual services provided at CTCs are the same regardless of where the client tested, clients coming from mobile/outreach sites experienced more delays at several stages, reflecting the additional challenges of travelling to a new facility with unfamiliar schedules. Table 3 summarizes the processes and timelines at CTCs, comparing clients diagnosed at facility-based and mobile sites, whilst Table 4 summarizes the similarities and differences between the two models of testing.

**Table 4:** Summary of Similarities and differences in facility-based and mobile/outreach sites

	Facility-based site	Mobile/outreach sites
General		
Study working definitions	Facility-based testing sites: fixed or static facilities within the public or mission health sector (e.g. hospitals, health centres or dispensaries) where individuals walk in for HIV testing or other health care services	Outreach HIV testing sites include but are not limited to mobile testing clinics using cars or tents, home visits, workplaces, schools, campaigns or special event testing services like World AIDS Day Most mobile/outreach testing sites are operating under Non-Governmental Organizations
Funders	Government facilities	Non-Governmental organizations/sponsors
Client/individual level		
Reason for testing at a particular site	Coming voluntarily for HIV test, or Coming for medical care and HIV test suggested by staff (PITC), or Referred from lower level facilities, or Admitted to the ward and then advised to take a test	People come for HIV testing because they were passing by and saw the tents of track and decide to test, or Heard the public announcement for free services in their area, or Coming voluntarily for HIV test because HIV testing services were brought closer to where they live
Number of clients tested during the study period	5234 with 21.5% diagnosed HIV positive	6539 with 7.9% diagnosed HIV positive
Health care provider level		
Human resource or types of staff available	Nurses: 1–2 in VCT Nurses 2–5 at CTC Doctors 1–2 doctors at CTC Other staff- lab technician, pharmacy, and recorders 1–4	Nurse counsellors 3–6 Home-based carers 1–2 Doctors only at the research mobile Mobile sites normally have more counsellors, home-based carers (HBCs), and peer health educators; they only have doctors in special events
Client follow up	PITC actively implemented but no active post-test follow-up of clients Some minimal follow-up for clients who are initiated on ART by treatment experts (in facilities where they exist) to ensure that they do not stop treatment	KIHUMBE, SHDEPHA, and St John Hus sites follow up their HIV positive clients through the Home-based care workers who are paid by the organization HBCs sometimes assist clients with the linkage to care process and conduct home visits for support and follow up HBC provides the numbers of the clients who are lost to follow-up to the site manager who compiles and writes an annual report for loss to follow-up and sends reports to the regional office
System level		
HIV testing and HIV care activities	The sites offer HIV testing and HIV care services Clients come from home following services at the health facility Clients may come to the site for another sickness, and the health care provider may advise the person to test for HIV	The sites offer HIV testing and refer the clients to facility-based CTCs for HIV care services Conducts mobile/outreach HIV testing, moves from one place to another following people in the community or their homes Some of the mobile sites (e.g. KIHUMBE) also conduct HIV test in some of their offices in fixed buildings During special events and campaigns, music and dancing groups entertaining people are standard
HIV testing place/venue	Fixed buildings like hospitals, health centres or dispensaries (at the HIV testing and CTC section) In one facility, there was a television set at the CTC clinic clients were watching while waiting for services	Mobile cars/vans, Fixed buildings/clinics Open areas/grounds Tents
Functioning tools/equipment and guidelines	Register books Reports Referral form/letter Patient files Laboratory services available Guidelines- HIV testing guidelines were not available at the site, but most staff reported being trained on the current guidelines	Register books Reports Referral forms Clients tracing forms for HBCs No laboratory services except for the research mobile HIV testing guidelines were not available at the site, but most staff reported being trained on the current guidelines
Record keeping	Register books and monthly reports in some sites were incomplete	All register books and monthly reports were well kept and complete
Information available	Health education on HIV and HIV treatment adherence sessions Posters related to HIV diseases fixed in waiting areas	Advertisements of services by local radio stations Car announcement/public announcements Campaigns Flyers with HIV information distributed to community members Community mobilization and invitation for free HIV testing
Testing algorithms	Use of Determine ™ as primary test if reactive Uni-gold is used for confirmation, However, Elisa test can be done if necessity arises	Use of Determine ™ as a primary test if reactive Uni-gold is used for confirmation If necessary, they refer clients for Elisa at the nearby hospital or ask the participants to come again after 2 weeks for re-testing
Referral procedure for HIV positive clients Same referral forms are used at the facility-based and mobile sites	Provide referral form to the CTC within the facility	Provide the referral letter/form and advise the client to link at the facility of their choice
Contextual level		
Availability of HIV testing and HIV care services	HIV testing services availability is better compared to availability of HIV care service In most of the sites (including dispensaries) HIV testing is done, although the distance was still a challenge especially in Chunya and Mbeya rural districts	HIV testing services are brought closer to clients’ homes, however, the challenge was to get care and treatment services In some cases, clients needed money for transport to reach a site where they can receive care

## Discussion

This study sought to describe and compare the processes and dynamics of HIV testing and linkage to care among newly-diagnosed individuals testing at facility-based and mobile/outreach sites in rural settings of Mbeya, Tanzania. We have compared the populations accessing HIV testing at mobile/outreach versus fixed/facility-based testing sites in rural settings of Mbeya and described the procedures of linkage and the roles of various actors including patients, providers, and clinic organization in arriving at successful linkage to care, in relation to national guidelines. Identification of characteristics of people, strengths and weaknesses in each model, and the capacities of the overall health system will help strengthen provision of universal testing and linkage to care for HIV-positive individuals [5, 10, 13, 39].

### HIV testing and management guidelines

Both mobile and facility-based HIV testing sites and HIV-CTCs attended to the clients in accordance with the National guidelines for HIV testing and HIV management. Although most sites did not have the paper/book guidelines in place, the staff had received training on the guidelines through seminars/workshops and understood their responsibilities with regard to HIV testing and care services. After HIV-positive diagnosis and post-test counseling, the care providers explained the available options for further care and provided the clients with referral letters. The difference was seen in the HIV testing sites: the mobile sites were actively following people up in their homes, and HIV testing was done out of hospital premises, with some activities that attracted more people to come for testing, while at the facility-based sites testing was passive and sometimes by PITC approach. Processes and procedures in the HIV care section were similar for all clients regardless of where they had tested, although those tested at the MMRC mobile site did not have to re-check their CD4 count test. At the CTC, the processes and procedures corresponded to what is reported in other studies [18, 19, 40]. At the time of the study, treatment was limited to patients with CD4 counts below 350 or with stage III or IV of HIV or certain coinfections.

### Shortage of healthcare workers in HIV care and treatment centres

The increasing rates of linkage to care found in our study compared to earlier research in Tanzania are highly encouraging, but staff shortages pose a significant challenge. We noted overcrowding and long waiting periods at the CTC, representing a further barrier to linkage to care to those clients who did follow up after a positive HIV test result and tried to register at a CTC. In Tanzania, only 52% of the government-identified required numbers of health workers are actually available in the health sector, a situation now considered a national crisis requiring continuous and collaborative attention [1].

Health worker density in Tanzania ranges from 4/10,000 population to 10/10,000 population [1]. All of the HIV CTCs included in this study had many clients while the number of staff was low, causing HIV-positive individuals to spend a long time at the clinic waiting for services. This is likely to affect the engagement and retention of patients in HIV care, as reported in other studies [9, 41, 42]. This is particularly challenging for remote areas like the study site, as most of the staff prefer to work in urban areas rather than rural areas with poor working and living environments. Plans are underway to increase the number and capacity of health and social welfare workers at all levels and areas of the country by 2018, and to reduce the shortage of staff from 52% in 2014 to 30% in 2019 [1].

### Choosing where to test

The study supports findings reported elsewhere that outreach testing methods increase rates of HIV testing, but linkage to care after testing positive is lower in the outreach testing approaches [13, 26]. This study found that more people tested at the mobile/outreach sites compared to the facility-based sites. The significantly lower rates of positive HIV test results in this population suggests that most people may be hoping for and expecting confirmation that they are HIV negative, in contrast to both the higher HIV rates and the respondent reports of testing because they felt sick at the facility-based sites. This finding supports the results of our cohort study, where many participants said they went to the facility–based site because they were sick [29]. In the mobile sites, services are provided by care providers who are not from within the community, and our respondents report that people therefore feel their results will remain confidential. This is likely particularly important for individuals who fear that they may learn that they have a positive test result. However, the problem for linkage to care occurs when the client is asked to go for HIV care at the same clinic they were initially avoiding for the testing, echoing similar findings and reasons for choosing to test at outreach services reported in other studies conducted in sub-Saharan African countries [43–45].

### Dynamics of HIV testing and linkage to care

The study revealed that different factors at the individual, provider, health system and contextual levels may influence the individual’s decision for testing, choices of testing site, and ultimately linkage to care and initiation of ART. The study revealed that mobile/outreach sites increased HIV awareness and uptake of voluntary HIV testing, particularly amongst younger and single adults, who are at greater risk of HIV infection—and may also be less likely to link to care. However, challenges remain with linking HIV-positive clients to care. The current referral processes in Tanzania need to be revisited to allow more active follow-up and assisted linkage to care, as this has been shown to enhance linkage in other areas [46, 47].

In addition to the importance of patient-level factors, our findings also reflect the positive impacts of well-implemented PITC on both testing and linkage to care.

At the system or program level, this study reveals some of the tradeoffs between policy objectives of maximizing testing versus maximizing linkage to care. Health systems research suggests that processes from HIV testing to linkage into HIV care may be different in health facility-based and outreach testing models, especially with respect to active referral to help individuals diagnosed with HIV to gain access to treatment and care services [48]. This study found that while mobile and outreach sites do indeed reach more people and actively try to facilitate linkage to care after an HIV positive test result more than facility-based sites do, the rates and timeliness of actual linkage to care are longer than for those testing at facility-based sites. This is the case despite the additional access barrier posed by overcrowding and long waiting times once clients arrive at CTCs to attempt to link to care. The mobile/outreach sites were not offering HIV care services. The presence of services in a site, together with standard referral procedures and staff who are motivated to offer and facilitate PITC, appears to outweigh the inconvenience of having to return several times for different stages of the care process, at least for individuals who make it to a facility-based site for an HIV test. This echoes findings reported by other linkage to care studies [26, 42, 49], suggesting that while specific contextual factors must be addressed to remove barriers to both testing and linkage to care in any given site, cross-cutting systemic barriers and facilitators occur across settings.

Improvements and streamlining in clinic organization (such as not requiring patients to return multiple times to give a blood sample for a CD4 count, obtain the CD4 count result, be staged and assessed for ART and treatment adherence sessions) would probably further enhance linkage to care. Our study highlights the importance of the system-level factors of availability of services and standard referral and care management procedures. Combined with provider-level adherence to PITC guidelines even in the absence of “going the extra mile” to support patients, these system-level factors facilitate high rates of timely linkage to care for most patients. What has not yet been addressed, however, is what it would take to reach the patients who do not come to HIV care facilities, and who face barriers of stigma, shock at a positive HIV test result, and distance to link to care. Our findings here and in previously published work [29] show that this is a much smaller proportion of the population living with HIV than just some years ago in rural Tanzania (where linkage to care within 4 months was only 14% [25], but there is still a long way to go to reach the 90–90–90 goal and ending AIDS by 2030.

### Strengths and limitations

Process evaluation of this nature determines whether program activities have been implemented as intended. The strength of this study is that it tracks in a stepwise manner the various actions or procedures from HIV testing to linkage into HIV care for newly HIV diagnosed clients, using multiple data sources and methods to triangulate data. A key limitation of this study is that because all of the randomly selected facility-based sites in our sample also had CTC facilities within the campus, we were not able to assess how linkage to care from facility-based sites without CTCs compares to linkage from mobile sites or from facilities offering comprehensive services within a single facility. The linkage rates controlling for patient characteristics were also not assessed in this study. A further limitation of the study is that, while collecting information on the process and procedure of linkage to care, we did not have access to those individuals who tested HIV-positive but were lost to follow-up, and we were unable to know if they had linked or not.

## Conclusion

In this study, understanding the populations accessing HIV testing at mobile or outreach and fixed/facility-based testing sites in hard-to-reach rural settings of Mbeya region, Tanzania, and comparing processes and dynamics from testing to linkage to care between these two testing models helped to generate useful program-relevant information. The national guidelines for HIV testing and HIV management were observed by the health care providers in both models. Despite the more proactive care and clients’ appreciation of confidentiality at the mobile sites, clients who tested at facility-based sites were more likely to link to care and to do so sooner because of integration of HIV testing and HIV care services in the same location/spot, as well as that the clients sought HIV testing services because they were sick, and therefore had the desire to start treatment. People seeking testing at facility-based sites are thus likely to be more “treatment ready”—or “linkage ready”—than those seeking testing at mobile/outreach sites. This suggests the importance of considering a combination of patient-level factors, notably different reasons for testing and stigma, and well-established procedures and routines for each step between testing and initiation of treatment at all types of HIV counselling, testing and care facilities. Drawing on and further developing existing frameworks and approaches to access to care, this study’s findings may inform the development and adaptation of strategies that can respond to the challenges of newly HIV diagnosed individuals and be responsive to the health system realities.

## References

[1] MOHsw. Human resource for health and social welfare, Tanzania. 2014. https://www.jica.go.jp/project/tanzania/006/materials/ku57pq00001x6jyl-att/HRHSP_2014-2019.pdf. Accessed at 22 Aug 2017.

[2] UNAIDS.The Gap Report. Geneva: UNAIDS; 2014. www.unaids.org/en/media/unaids/contentassets/…/UNAIDS_Gap_report_en.pdf. Accessed 04 July 2017.

[3] UNAIDS. FAST-TRACK ending the AIDS epidemic by 2030. http://www.unaids.org/sites/default/files/media_asset/JC2686_WAD2014report_en.pdf. Accessed on 22 Nov 2017.

[4] Haber N, Tanser F, Bor J (2017). From HIV infection to therapeutic response: a population-based longitudinal HIV cascade-of-care study in KwaZulu-Natal, South Africa. Lancet HIV.

[5] Ostermann J, Reddy EA, Shorter MM (2011). Who tests, who doesn’t, and why? Uptake of mobile HIV counseling and testing in the kilimanjaro region of Tanzania. PLoS ONE.

[6] NACP. National AIDS Control Program in Tanzania. 2012. https://aidsfree.usaid.gov/sites/default/files/hts_policy_tanzania.pdf. Accessed 13 Apr 2017.

[7] MOH. National Comprehensive Guidelines for HIV Testing and Counselling, 2013.https://aidsfree.usaid.gov/sites/default/files/htc_tanzania_2013.pdf. Accessed 16 Oct 2017.

[8] Kapologwe N, Kabengula J, Msuya S (2011). Perceived barriers and attitudes of health care providers towards Provider-Initiated HIV Testing and Counseling in Mbeya region, southern highland zone of Tanzania. Pan Afr Med J..

[9] Layer EH, Kennedy CE, Beckham SW (2014). Multi-level factors affecting entry into and engagement in the HIV continuum of care in Iringa, Tanzania. PLoS ONE..

[10] Njau B, Ostermann J, Brown D (2014). HIV testing preferences in Tanzania: a qualitative exploration of the importance of confidentiality, accessibility, and quality of service. BMC Public Health.

[11] Cawley C, Wringe A, Todd J (2015). Risk factors for service use and trends in coverage of different HIV testing and counselling models in northwest Tanzania between 2003 and 2010. Trop Med Int Health.

[12] AVERT. HIV and AIDS in Tanzania, [Online] (2018). https://www.avert.org/professionals/hiv-around-world/sub-saharan-africa/tanzania. Accessed 6 Nov 2018.

[13] Hatcher AM, Kwena Z, Johnson MO (2013). Counseling and testing in rural Kenya. AIDS Behav.

[14] Rio C. Cascade of Care and its relevance to seek, test, treat and retain strategy. 2011. http://www.apa.org/about/gr/issues/substance-abuse/del-rio-nida.pdf. Accessed 12 Jun 2017.

[15] Dombrowski J. Linkage to care case study. 2013; 6–25. depts.washington.edu/nwaetc/presentations/uploads/109/linkage_to_care.pdf. Accessed 12 Jun 2017.

[16] Philbin MM, Tanne AE, DuVal A (2015). Factors affecting linkage to care and engagement in care for newly diagnosed HIV-positive adolescent medicine clinics in the United States. AIDS Behav.

[17] Ulett KB, Willig JH, Lin H (2009). The therapeutic implications of timely linkage and early retention in HIV care. AIDS Patient Care STDS..

[18] Gerdts S, Wagenaar B, Farguhar C (2014). Linkage to HIV care and antiretroviral therapy by hiv testing service type in central Mozambique: a retrospective cohort study. J Acquir Immune Defic Syndr.

[19] Haber N, Tanser F, Naidu K, et al. HIV System Assessment with Longitudinal Treatment Cascade in KwaZulu-Natal, South Africa, http://scholar.harvard.edu/noahhaber/publications/hiv-system-assessment-longitudinal-treatment-cascade-kwazulu-natal-SouthAfrica. In: 19th International workshop on HIV observational databases. Sicily; 2015. p. 34.

[20] Bhatia R, Giordano TP (2011). Persons newly diagnosed with HIV infection are at high risk for depression and poor linkage to care: results from the steps study. AIDS Behav.

[21] Keller SC, Yehia BR, Eberhart MG (2013). Accuracy of definitions for linkage to care in persons living with HIV. J Acquir Immune Defic Syndr.

[22] Rosen S, Fox MP (2011). Retention in HIV care between testing and treatment in sub-saharan Africa: a systematic review. PLoS Med.

[23] Bassett IV, Regan S, Luthuli P (2013). Linkage to care following community-based mobile HIV testing compared with clinic-based testing in Umlazi Township, Durban, South Africa. HIV Med.

[24] Sharma M, Ying R, Tarr G (2015). A systematic review and meta-analysis of community and facility-based approaches to address gaps in HIV testing and linkage in sub-Saharan Africa. HHS Public Access.

[25] Nsigaye Wringe A, Roura M (2009). From HIV diagnosis to treatment: evaluation of a referral system to promote and monitor access to antiretroviral therapy in rural Tanzania. J Int AIDS Soc..

[26] Bassett I, Regan S, Luthuli P (2014). Linkage to care following community-based mobile HIV testing compared with clinic-based testing in Umlazi Township, Durban, South Africa. HIV Med.

[27] Labhardt ND, Ringera I, Lejone TI (2016). Same day ART initiation versus clinic-based pre-ART assessment and counselling for individuals newly tested HIV-positive during community-based HIV testing in rural Lesotho—a randomized controlled trial (CASCADE trial). BMC Public Health.

[28] Simmelink A. High uptake but low rates of linkage to care following home-based integrated HIV voluntary counseling and testing services and non-communicable disease screening in Ifakara, Tanzania. 2014. http://scripties.umcg.eldoc.ub.rug.nl/root/geneeskunde/2014/SimmelinkAM/. Accessed 05 July 2017.

[29] Sanga ES, Lerebo W, Mushi AK (2017). Linkage into care among newly diagnosed HIV-positive individuals tested through outreach and facility-based HIV testing models in Mbeya, Tanzania: a prospective mixed-method cohort study. BMJ Open..

[30] Creswell JW (2015). A concise introduction to mixed methods research.

[31] Hulscher ME, Laurant MG, Grol RP (2003). Process evaluation on quality improvement interventions. Qual Saf Health Care.

[32] Friese S. ATLAS. ti 7 User Guide and Reference. 2013. http://atlasti.com/wp-content/uploads/2014/05/atlasti_v7_manual_201312.pdf?q=/uploads/media/atlasti_v7_manual_201312.pdf. Accessed 15 May 2016.

[33] Miles M, Huberman M (1994). An expanded source book: qualitative data analysis.

[34] Govindasamy D, Ford N, Kranzer K (2012). Risk factors, barriers and facilitators for linkage to antiretroviral therapy care: a systematic review. AIDS.

[35] Hodgson I, Plummer ML, Konopka SN (2014). A systematic review of individual and contextual factors affecting ART initiation, adherence, and retention for HIV-infected pregnant and postpartum women. PLoS ONE..

[36] MOHsw. National Guideline for Management of HIV and AIDS 5th Edition-CIRcular of Revised guideline for management of HIV/AIDS. www.nacp.go.tz/site/download/nationalguideline2012.pdf. Accessed 15 Jun 2016.

[37] Tong A., Sainsbury P., Craig J. (2007). Consolidated criteria for reporting qualitative research (COREQ): a 32-item checklist for interviews and focus groups. International Journal for Quality in Health Care.

[38] Sanga ES, Lerebo W, Mushi AK, et al. Linkage into care among newly diagnosed HIV positive individuals tested through outreach and facility-based HIV testing. Linkage into care among newly diagnosed HIV-positive individuals tested through outreach and facility- based HIV testing models in. 2017. p. 1–10. 10.1136/bmjopen-2016-013733 PMC5541440 28404611

[39] Govindasamy D, van Schaik N, Kranzer K (2011). Linkage to HIV care from a mobile testing unit in South Africa by different CD4 count strata. J Acquir Immune Defic Syndr.

[40] Genberg BL, Naanyu V, Wachira J (2015). Linkage to and engagement in HIV care in western Kenya: an observational study using population-based estimates from home-based counselling and testing. Lancet HIV.

[41] Labhardt Niklaus Daniel, Motlomelo Masetsibi, Cerutti Bernard, Pfeiffer Karolin, Kamele Mashaete, Hobbins Michael A., Ehmer Jochen (2014). Home-Based Versus Mobile Clinic HIV Testing and Counseling in Rural Lesotho: A Cluster-Randomized Trial. PLoS Medicine.

[42] Wachira J, Naanyu V, Genberg B (2014). Health facility barriers to HIV linkage and retention in Western Kenya. BMC Health Serv Res.

[43] MRACP. Mbeya Regional AIDS Control Programme. Mbeya Regional AIDS Control Program-Annual Report. 2012.

[44] Meehan S-A, Leon N, Naidoo P (2015). Availability and acceptability of HIV counselling and testing services. A qualitative study comparing clients’ experiences of accessing HIV testing at public sector primary health care facilities or non-governmental mobile services in Cape Town, South Afr. BMC Public Health.

[45] Bassett IV, Regan S, Mbonambi H (2015). Finding HIV in hard to reach populations: mobile HIV testing and geospatial mapping in Umlazi Township, Durban. South Africa. AIDS Behav..

[46] Ware NC, Wyatt MA, Asiimwe S, et al. How home HIV testing and counselling with follow-up support achieves high testing coverage and linkage to treatment and prevention : a qualitative analysis from Uganda. 2016. p. 1–7. 10.7448/IAS.19.1.20929 PMC4928103 27357495

[47] Kiene SM, Kalichman SC, Sileo KM (2017). Efficacy of an enhanced linkage to HIV care intervention at improving linkage to HIV care and achieving viral suppression following home-based HIV testing in rural Uganda. BMC Infect Dis.

[48] Stuart L, Harkins J, Wigley M. Establishing referral networks for comprehensive HIV care in low-resource settings. 2005; 1–17. http://pdf.usaid.gov/pdf.docs/Pnadf677.pdf. Accessed 17 July 2017.

[49] Medley A, Ackers M, Amolloh M (2013). Early uptake of HIV clinical care after testing HIV-positive during home-based testing and counseling in western Kenya. AIDS Behav.

